# Immune manifestations with checkpoint inhibitors in a single Brazilian center: experience and literature review

**DOI:** 10.2144/fsoa-2020-0129

**Published:** 2020-11-23

**Authors:** Rafael A Schmerling, Antonio C Buzaid, Carolina K Haddad, Fabio AB Schutz, Fabio R Kater, Juliana Pimenta, Fernando Maluf, William N William, Camila Lopes, Dafne R Bromberg, Ana C Oliveira, Ricardo Golmia, Morton Scheinberg

**Affiliations:** 1 Oncology Center & Center for Autoimmune Diseases at Hospital A Beneficência Portuguesa, São Paulo, Brazil

**Keywords:** autoimmune disease, checkpoint inhibitors, endocrine system, immunotherapy, malignant melanoma, tumor

## Abstract

**Objectives:**

The presence of autoimmune events were recorded in patients receiving immune checkpoint inhibitors.

**Materials & Methods:**

Retrospective study in patients receiving immune checkpoint inhibitors (ICIs) during the period of 2012–2019.

**Results:**

A total of 554 patients received ICIs of which 123 developed an immune related adverse event. Twenty one (17%) with toxicity were identified as having a pre-existing autoimmune disease and 88 required treatment with corticosteroids or hormone replacement. Thirty two (26%) out of 123 had to temporarily discontinue ICIs due to autoimmune manifestations. Endocrine and skin manifestations were the most prevalent immune disorders in our cohort. In melanoma better efficacy was seen in patients with immune toxicity.

**Conclusion:**

Autoimmune diseases appear in patients receiving ICIs in this real world experience. Our results differ from other series on the frequency of autoimmunity. Complete discontinuation of ICIs due to autoimmunity was rare.

## Lay abstract

The development of autoimmune events in patients receiving the modern forms of cancer immunotherapy are not unexpected since the presence of robust immune response against tumors relies on mechanisms of T-cell activation and release of cytotoxic cytokines that can also mediate autoimmune inflammatory conditions. In fact, some studies including ours points to better efficacy against tumor in patients that also develop autoimmune toxicity. Cancer therapy has now five consolidated forms of treatment, Chemotherapy, Radiotherapy, Surgery Biologic Therapy and second generation Immunotherapy.

Immune checkpoint inhibitors (ICIs) such as monoclonal antibodies against cytotoxic T lymphocyte antigen-4 (CTLA-4), programmed cell death (PD-1), and the programmed death-ligand (PD-L1) are effective against several cancers, such as melanoma, non-small-cell lung carcinomas, renal cell carcinomas, and Hodgkin's lymphoma, amongst others [1–3]. However, ICIs widespread use has been associated with the development of immune events, a toxicity expected since checkpoints are involved in regulating self-tolerance and pathophysiology of autoimmune manifestations [4,5]. The Oncology Center of the Hospital A Beneficência Portuguesa, São Paulo, is a pioneer in Brazil on the use of ICIs since 2012, when research trials were followed by the clinical practice of applying ICIs. In this study we reviewed the incidence and the most frequent organ autoimmune manifestations observed in our institution in the real world when patients were treated with single agents or combination therapy and compared these results with the experience described by other oncology services.

## Materials & methods

We collected data from 547 patients who received ICIs from the start of 2012 until mid-2019. The study was approved by the institutional review board of Hospital A Beneficência Portuguesa in São Paulo, Brazil. Retrospectively, data were collected on sex, age, tumor type, name and class of ICI applied and the type of immune manifestations detected for each patient and pertinent associated clinical findings such as time for the first immune event, the outcome of the autoimmune manifestations, correlation with tumor response and the presence of immune adverse events. An update on the current published literature was conducted using articles indexed in Pubmed, Medline, and Embase using the following keywords, checkpoint inhibition therapy, autoimmune diseases, tumor immunotherapy, monoclonal antibodies and checkpoint inhibitor. Our findings were compared with published data from other oncology services and discussed accordingly. Autoimmune events defined as toxicity was defined by grades 1 to 4 as outlined by Villadolid J *et al.* [6] Statistical comparisons were performed by the Chi-square test.

## Results

Patient demographics, ICI used and the tumor distribution are presented on Table 1. In total, more than 50% of the patients were men and 34% had malignant melanoma. Nivolumab, Pembrolizumab and Ipilimumab accounted for the majority of ICIs and the mean number of months for the appearance of autoimmunity event was 5 (range: 2–7). Patients receiving Ipilimumab took more months to develop toxicity (7 months) than patients receiving the combination of Ipilimumab and Pembrolizumab (2 months) or only an anti-PD-1 (5 months). Nivolumab, Pembrolizumab and Ipilimumab accounted for 98% of the immune-related adverse events (irAEs) observed in our center. The total number of drugs used in treatment are shown on Table 1. The total number of patients is smaller than the number of patients receiving ICIs since there were patients that received more than one ICIs as single agent or in combination. Combinations seen in patients with irAE were Pembrolizumab with Ipilimumab and Nivolumab with Ipilimumab.

**Table 1.** Demographics identification of checkpoint used and tumor distribution in patients with irAE.

	Patients with irAE	Patients without irAE	Total
**Demographic**			
Men	76	264	547
Women	47	160	
**Treatment**			
Atezolizumab	2	55	57
Avelumab	1	2	3
Durvalumab	1	5	6
Ipilimumab	37	82	119
Nivolumab	60	228	288
Pembrolizumab	33	125	158
**Underlying diseases**			
	63 melanoma	125 melanoma	188
	60 others	299 others	359
**Pre-existing autoimmune condition**			
	21	Not mentioned	

irAE: immune-related adverse events.

**Figure 1.**
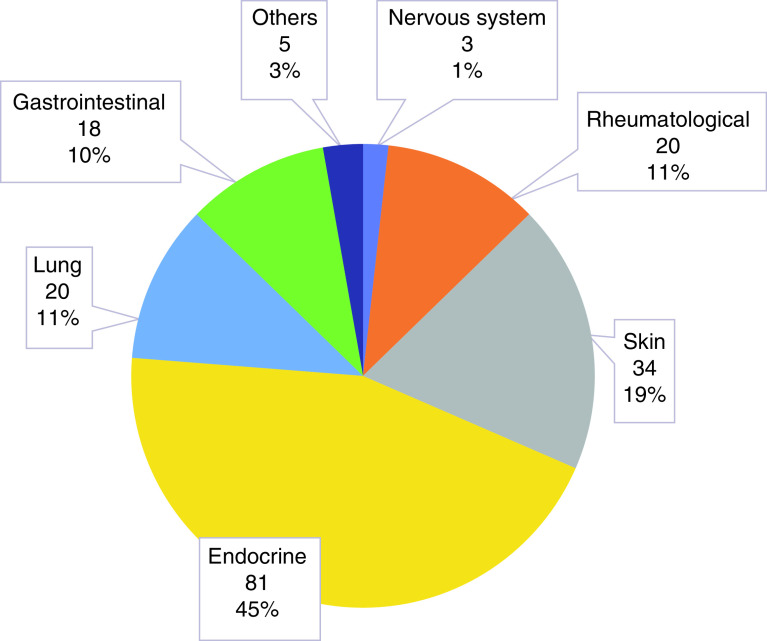
Autoimmune manifestations. Frequency (%) applies to the total number of autoimmune manifestations (n=181).

Autoimmune manifestations and frequency are highlighted in Figure 1, with endocrine and skin, showing the greater incidence followed by lung, rheumatological, gastrointestinal, and neurological findings. In Table 2, we provide the frequency of toxicity in which 80% were grades 1 and 2, accounting for the majority of irAEs. Twenty percent of the patients had toxicity grades 3 or 4 that led to therapy withholding or switching to other ICI.

**Table 2.** Degree of toxicity according to the respective grades in the patient population with irAE.

G1	G2	G3 / G4
37%	43%	20%

irAE: immune-related adverse event.

The respective therapy for the irAEs is shown in Table 3. Of note, few patients required therapy apart from corticosteroids and hormone replacement. The outcome of the toxicities (Table 4) show that most of the patients (55%) continued their treatment with respective ICIs despite the immune adverse event. Withdrawal was more frequently seen with Pembrolizumab followed by Nivolumab and Ipilimumab accounting for approximately one quarter of the patients. Table 5 presents distribution of toxicities by organ and class of ICIs used in our center. Anti-PD-1 was most often associated with irAES followed by anti-CTLA-4. Endocrine was the most common type of immune manifestation with anti-PD-1, followed by anti-CTLA-4 and the association of both. However, with anti-PD-L1 the majority of the events were skin manifestations, although this was not a frequent prescription to our cancer patients.

**Table 3.** Treatment of toxicity in the patient population.

ICIs	Patients with steroids (n)	Patients with hormone (n)	Patients with biologic therapy (n)
Atezolizumab	2	-	-
Avelumab	1		
Durvalumab	-	1	
Ipilimumab	10	9	
Nivolumab	21	17	2
Pembrolizumab	12	7	3
Combination	4	4	1
**Total (%)**	Steroids 53%	Hormone 40%	Biologic 7%

ICI: Immune checkpoint inhibitor.

**Table 4.** Outcomes of treatment with immune checkpoint inhibitors and irAEs: n of patients (%).

Withdrawn because of toxicity	Withdrawing for other reasons	Continuation	Changed ICIs
32	9	68	14
(26%)	(7%)	(55%)	(11%)
Of those: Pembrolizumab 40% Nivolumab 28% Ipilimumab 16%			

ICI: Immune checkpoint inhibitor; irAEs: Immune-related adverse events.

**Table 5.** Class of immune checkpoint inhibitors and organ manifestation.

	PD-1	CTLA-4	PD-L1	PD-1 + CTLA-4
Nervous system	-	-	-	3
Rheumatologic	13	3	1	2
Skin	24	6	2	3
Endocrine	50	21	1	8
Lung	17	1	-	1
Gastrointestinal	10	5	-	5
Others	5	-	-	-
Total	119	36	4	22

CTLA-4: Cytotoxic T lymphocyte antigen-4; PD-1: Programmed cell death; PD-L1: Program death ligand.

It is known that patients receiving ICIs with previous autoimmune diseases could have reactivation of their associated underlying disease. Pre-existing autoimmune diseases with exacerbation during ICIs therapy was seen in 21 patients of the total patient population with irAES. Endocrine and skin autoimmune diseases were seen at a higher frequency in cases of exacerbation and are listed in Table 6. Sixty-three patients of a total of 188 patients (33%) with malignant melanoma had irAEs and, 19 (30%) died during follow-up. Of the 125 melanoma patients without irAEs, 55 (44%) died in the same period. The mortality rate difference was statistically significant (p = 0.025) between those with toxicity and those without. From this data, there is a suggestion that the occurrence of irAEs may indicate a better efficacy. However, this comparison should be seen with reservations since 15 patients with toxicity and 43 without were lost on follow-up and could not be listed for comparison.

**Table 6.** Pre-existing autoimmune disease that had exacerbations on immune checkpoint inhibitor therapy.

Arthritis	1
Hypothyroidism	9
Vitiligo	4
Diabetes	4
Pemphigus	1
Psoriasis	2
**Total**	**21**

## Discussion

Cancer immunotherapy through ICIs is a breakthrough for the treatment of cancer. The fact that checkpoint therapies can induce the appearance of autoimmune manifestations is not unexpected since there is extensive data available from knockout mice for CTLA-4 that develop a rapidly progressive lymphoproliferative autoimmune disease and CTLA-4 gene polymorphisms have been associated with an increased risk of autoimmune disease. However, in real life autoimmune diseases became more evident as clinical trials for the most part have excluded preexisting autoimmune diseases [7,8]. In this study, we performed a retrospective medical record review on autoimmune diseases in patients with cancer treated with immune checkpoint inhibitors in our service since its introduction in 2012 and compared the findings with what has been reported by publications from multicenter or single centers. The percentage of any grade of autoimmune toxicity in our center was 22%. This finding is within the range reported in similar studies. However, there is considerable variation in the published literature with some studies pointing to a higher incidence up to 60%, particularly when nonspecific gastrointestinal symptoms of short duration like one-week diarrhea, vomiting and abdominal pain were listed, not truly reflective of an immune inflammatory disease. In our center anti-PD-1 had a higher association with toxicity compared with anti-PD-L1 and anti-CTLA-4. Also, it also appears that the variation on toxicity depends on the organ predominantly specified, but taken all reports available, the percentage observed in our cohort was less than 30% and within the range of papers published in the literature [9,10]. One of the variables that we believe may not be present in all studies is that in our center, experienced physicians could identify and perform early diagnosis of an irAE. The majority of toxicities in our series appeared as early as 2 months usually with endocrine manifestations, and they could be seen as far seven months from initiation of therapy.

## Organ specific autoimmunity

Autoimmune organ manifestations detected in our center series were (in the order of frequency) first the endocrine system followed by the skin, pulmonary, musculoskeletal, gastrointestinal, and nervous systems and in isolated cases, other organs. Contrary to what has been found in other reports, endocrine dysfunction, in our cohort, was the most frequent autoimmune manifestation, identified in 81 patients. Second, in occurrence, was the skin with 34 patients followed by rheumatic, pulmonary, gastrointestinal, and neuroimmune disturbances. In the series from the city of São Paulo, hypothyroidism was frequent (53%), hyperthyroidism was rare, followed by signs of toxicity in the hypothalamic-pituitary-adrenal axis indicated by hypophysitis (16%). However, isolated autoimmune adrenocorticotropic hormone deficiency was identified in five out of 81 patients with endocrine autoimmunity [11–13]. Type 1 diabetes was also observed but was uncommon; however, some have reported this disease more frequently. Our endocrine findings are consistent with other reports from specialized centers, mainly when the respective incidences are extracted from the total number of autoimmune diseases rather than from the total number of patients receiving ICIs, where other organ involvement such as the skin is reported with a higher incidence. Endocrine toxicities tend to be permanent and frequently require lifelong hormone replacement; thus, an endocrine consultation should be sought. However, this contrasts with most other autoimmune manifestations that tend to be transient and reversible and respond to therapy. Dermatological autoimmune skin manifestations were reported in other series as the most frequent clinical toxicity in the ICIs related autoimmune spectrum, which was uncommon at our center. Up to 50% of patients receiving checkpoints inhibitors experienced skin rashes, sometimes pruritic and reversible, and they were not considered autoimmune, but rather allergic, that tended to be mild and not lead to treatment discontinuation. These skin symptoms usually occurred within the first 23 weeks after initiation of therapy. In our series, vitiligo was present in 23 patients, predominantly in melanoma patients, followed by chronic dermatitis (spongiotic eczema), psoriasis, and bullous pemphigus. The association between melanoma and the appearance of vitiligo, independent of ICIs, is well known and reported in about 20% of patients; and its increased occurrence with the use of ICIs, seen in our center, was previously reported. The association of melanoma with irAEs, observed in other series, was seen in our cohort representing 50% of all autoimmune events in patients with irAEs [14,15]. Pulmonary manifestations detected by symptoms and chest tomography were observed in 20 patients in our series. They were mild and treated conservatively with corticosteroids with no discontinuation observed. In our center, this incidence was about 11%, similar to that reported by other centers such as the Mayo Clinic [16]. Arthralgia and arthritis are the most commonly reported rheumatic symptoms, followed by myositis; in our series, arthralgia was more frequent than established arthritis observed in 15% of our cohort. Destructive arthritis and polymyalgia, seen in other reports, were not identified in our series. One patient was diagnosed with severe dermatopolymyositis, and one with Sjogren-Sicca disease. However, similar to other reports, these manifestations seem to persist longer than other autoimmune toxicities [17].

## Diarrhea & colitis

The presence of gastrointestinal complaints during cancer therapy is well-known. The same appears to occur with stool frequency and colitis with ICIs, and they can occur more in combination therapy. In our series, around 10% of the patients (18 patients from 123) had GI complaints, a somewhat smaller number from those reported in other studies that were as high as 30% in some series with established autoimmune inflammatory bowel disease that required biologic therapy [18]. However, in most of the series, it is unclear whether inflammatory autoimmune disease was present. In patients with Crohn's disease, the disease's exacerbation was reported using ICIs and appeared to be more frequent with CTLA-4 than with PD-1 and PD-L1 blockade [19].

## Neurological

Reported neurological complications are rare and quite diverse. Autoimmune neurological diseases are described by various authors, including encephalopathy, Guillain Barré neuropathy, multiple sclerosis-like, myasthenia gravis, transverse myelitis, and peripheral neuropathy in various series. The frequency of ICIs inducing neurological adverse events is about 1%. Our study has not seen an overlap of neurological symptoms with other autoimmune manifestations such as myasthenia gravis myositis, joint symptoms, and neuritis, but these symptoms were observed in other cohorts. Three patients in our series had well-documented autoimmune encephalitis, myasthenia gravis, and Guillain Barré acute neuropathy, which have been well-documented neurological autoimmune manifestations in patients receiving checkpoint inhibitors. Our experience resembles that of the literature [20,21]. In general, the exacerbation risks of previous autoimmune disease in patients receiving ICIs are a potential concern and reported in other series. In our center, this risk occurred and was observed in 21 patients. The conventional treatment with immunosuppression and hormone replacement was similar to other published reports, and discontinuation of therapy due to autoimmune disease was not a common occurrence in our series [22,23].

Identification of predictive biomarkers to most likely benefit from checkpoint blockade has been extensive, including molecular research and tumor-infiltrating lymphocytes. In our series, autoimmunity development was associated with good clinical response in melanoma patients, particularly with the appearance of vitiligo, but this was also seen with other tumors. Our experience confirms this observation in our melanoma cohort. Patients with autoimmune toxicity had better survival than patients without autoimmune toxicity, and our data also points to this conclusion [24–26]. Our study has several limitations given its retrospective design, risk of incomplete data collection, and variations in the documentation. Finally, the difference between actual adverse autoimmune events and an underlying autoimmune disease flare could not always be determined with enough confidence.

## Conclusion

Although it has been almost six years since checkpoint inhibitors were introduced in oncology, there are very few reports from the experience of its use and respective autoimmune toxicity in Latin America. Our findings showed that endocrine and skin are the most frequent autoimmune manifestations followed by lung and rheumatic in this large retrospective single-center study. Low-grade 1 and 2 adverse events were predominant in our series. Management with corticosteroids and immunosuppressive agents were successful in most patients; biologic therapy was used in a few refractory cases. Permanent discontinuation of ICIs due to irAE was rare. Malignant melanoma was the tumor in which irAEs occurred with the highest frequency. Autoimmunity in our series showed to be most frequent with PD-1, followed by CTLA-4 blockade. The use of anti-PD-1 in our report was most frequent with the appearance of endocrine reactions common. In our series, the use of anti-PD-L1 was not frequent in our cancer patients. With a few exceptions outlined in the discussion, our findings are similar to other retrospective reports on the topic from Europe and North America, including the observation that better tumor responses associate with those who experienced autoimmune toxicities [27–31].

## Future perspective

Checkpoint inhibitors in the next five years are likely to become first-line treatments in several tumors as combinations with traditional anticancer agents with ICIs continue to evolve. Second generation ICIs are expected to be more targeted or selective and possibly associated with fewer autoimmune development. Although our data and others suggest better efficacy of ICIs in those who experience autoimmune toxicities, these data need confirmation in prospective trials to validate this association. Our experience shows that anti-PD-1 agents to be more associated with toxicity; however, it is the ICIs mostly used in practice, and the advent of new combinations and second-generation anti-PD-1s may change this landscape. [32,33].

**Summary points**

- Cancer immunotherapy with checkpoint inhibitors (ICIs) has become standard therapy in several malignancies.
- ICIs can induce autoimmune manifestations, expected given its mechanism and experimental work, and confirmed in real-life practice.
- Thus far, the largest reported experience of the safety profile using checkpoint inhibitors in a Latin American Oncology Center is at Hospital A Beneficência Portuguesa in São Paulo, Brazil.
- Retrospective data were collected during seven years as it relates to the occurrence of autoimmune manifestations with the use of ICIs.
- The presence of toxicity with a respective drug or combination was aligned and paired with the respective organ manifestation and the presence of previous autoimmune disease.
- Toxicity was observed in 22% of the patients receiving ICIs.
- Endocrine and skin were the most common, followed by rheumatic and lung manifestations.
- In some patients, pre-existing autoimmune disease was aggravated and treated with conventional therapy, including corticosteroids, hormone replacement therapy, and, seldom, biologic therapy.
- Our findings are comparable to similar studies, although the frequency and incidence of organ manifestations in our report differ in part from other large series, particularly as it relates to gastrointestinal manifestations.
- Malignant melanoma was the tumor in which autoimmune toxicity occurred with the highest frequency.
- Our study's observations support that better tumor responses are seen in patients with the presence of immune toxicities.
